# Food prescribing in Canada: evidence, critiques and opportunities

**DOI:** 10.24095/hpcdp.44.6.04

**Published:** 2024-06

**Authors:** Matthew Little, Warren Dodd, Laura Jane Brubacher, Abby Richter

**Affiliations:** 1 School of Public Health and Social Policy, University of Victoria, Victoria, British Columbia, Canada; 2 School of Public Health Sciences, University of Waterloo, Waterloo, Ontario, Canada; 3 Guelph Community Health Centre, Guelph, Ontario, Canada; 4 The SEED, Guelph, Ontario, Canada

**Keywords:** food prescribing, food insecurity, dietary adequacy, chronic disease prevention, chronic disease management, food is medicine, social prescribing

## Abstract

**Introduction:**

There is growing interest in food prescriptions, which leverage health care settings to provide patients access to healthy foods through vouchers or food boxes. In this commentary, we draw on our experiences and interest in food prescribing to provide a summary of the current evidence on this intervention model and critically assess its limitations and opportunities.

**Rationale:**

Food insecurity is an important determinant of health and is associated with compromised dietary adequacy, higher rates of chronic diseases, and higher health service utilization and costs. Aligning with recent discourse on social prescribing and “food is medicine” approaches, food prescribing can empower health care providers to link patients with supports to improve food access and limit barriers to healthy diets. Food prescribing has been shown to improve fruit and vegetable intake and household food insecurity, although impacts on health outcomes are inconclusive. Research on food prescribing in the Canadian context is limited and there is a need to establish evidence of effectiveness and best practices.

**Conclusion:**

As food prescribing continues to gain traction in Canada, there is a need to assess the effectiveness, cost-efficiency, limitations and potential paternalism of this intervention model. Further, it is necessary to assess how food prescribing fits into broader social welfare systems that aim to address the underlying determinants of food insecurity.

**Highlights**

- Food prescribing is one of several “food is medicine” approaches that leverage health care interactions to address food insecurity and improve nutrition among patients.
- Food prescribing has been shown to improve fruit and vegetable intake and household food insecurity.
- There is a need to critically evaluate the effectiveness and cost-efficiency of food prescribing relative to other health care, public health and social welfare programs.

## Introduction

Food insecurity is a public health crisis in Canada. A wide body of literature links household food insecurity, defined as inadequate or insecure access to food due to financial constraints,^1^ to compromised dietary adequacy,^2^ higher rates of chronic diseases and infections,^3^ poorer mental health^4^ and premature mortality.^5^ Further, household food insecurity is associated with higher health service utilization and costs.^1^ Food insecurity is therefore an important social determinant of health that must be urgently addressed through poverty alleviation and public health measures. 

There is growing interest in leveraging primary health care settings to provide patients with better access to healthy foods to simultaneously address food insecurity and improve nutritional adequacy and health. “Food prescriptions” are an area of innovation and exploration, whereby health care practitioners identify patients who are food insecure or at risk of diet-related chronic diseases and provide them access to subsidized or free healthy foods.^6^ Food prescriptions align with recent calls for social prescribing, in which health care providers connect patients directly to nonclinical services to address social determinants of health and improve health and well-being.^7^ Food prescribing is one of several “food is medicine” approaches, alongside medically tailored meals and groceries.^8^

Food prescribing programs and research have rapidly gained popularity in the United States, driven by the 2018 Farm Bill, a federal bill that included USD 25 million of funding to implement and evaluate fruit and vegetable prescribing in health care.^9^ While the volume of food prescribing programs and research in the Canadian context is substantially lower, food prescribing models have been adopted and evaluated in several settings in Alberta^10^ and Ontario.^11^-^13^ Such models have been the subject of much discourse in media, academia and not-for-profits, underscoring the mounting interest in food prescribing. 

Our research team has collaborated with the Guelph Community Health Centre (CHC) since 2019 to implement and evaluate multiple phases of a fruit and vegetable prescription program called Fresh Food Prescription (FFRx).^11^ Drawing on our direct experience and keen interest in this intervention model, our aim is to provide a critical assessment of food prescribing in Canada.

## What is the current evidence on food prescribing?

Food prescribing interventions rely on health care practitioners (e.g. physicians, nurse practitioners and allied health professionals) to identify eligible patients and provide a food prescription, which often includes vouchers redeemable for fruits and vegetables, access to a nutritionist or dietitian, and/or food literacy programming (e.g. nutrition education, pamphlets, cooking classes, etc.).^14^ While there have been dozens of recent evaluations of food prescribing programs in the United States, there remains little consensus on impacts and best practices, and evidence in the Canadian context is limited. Many pre-post intervention studies (including ours) report improved fruit and vegetable consumption and reduced household food insecurity among recipients of food prescriptions.^6^,^14^ In one meta-analysis, pooled estimates revealed a 22% increase in fruit and vegetable consumption among recipients.^14^ However, evidence for the impacts of food prescribing on patient health outcomes is far less conclusive, with some studies reporting improvements in pre-post blood pressure,^15^,^16^ BMI^17^ and HbA1c (among people with diabetes),^18^ but many others reporting no measurable health improvements.^19^

Simulation studies suggest that implementing produce prescriptions may generate substantial health gains and be highly cost effective,^20^ but no studies have yet examined impacts on real-world health care utilization and spending. Furthermore, the evidence base on food prescribing is plagued by severe methodological limitations, including small sample sizes; limited intervention duration (usually <23weeks); incomplete outcome data; nonvalidated measurement tools; and nonrandomized study designs without a control or comparison group.^6^

## What is the state of food prescribing in Canada?

Despite limited evidence, interest in food prescribing is rapidly growing in Canada. Reasons for this sudden attention are multiple. Expansion of food prescribing discourse has paralleled the emergence of social prescribing more broadly. Examples of such initiatives include the Rx: Community pilot project of the Alliance for Healthier Communities (which supported 11 health centres across Ontario in initiating social prescribing projects) and the newly established Canadian Institute for Social Prescribing (CISP), which is anchored by the Canadian Red Cross and acts as a national knowledge-sharing hub. 

It is likely that Canadian health care providers, community organizations and researchers have gained inspiration from those in the United States, where an explosion of food prescribing has followed federal government investment since 2018.^9^ One can also see the attractiveness of food prescribing as a concept, which proposes a relatively simple solution—grounded in the familiarity and persuasiveness of “doctor’s orders”—to multiple crises, including food insecurity, dietary adequacy, nutrition-related chronic diseases and even planetary health.^21^ Support for food prescribing has also been driven by several not-for-profit agencies, including the Maple Leaf Centre for Food Security and Community Food Centres Canada. Despite this momentum, however, action on food prescribing in Canada remains nascent, with only a handful of (often temporary pilot) programs established and very few published research articles^11^,^13^ evaluating the impacts of programs. 

## A critical examination of food prescribing in Canada

It is likely that food prescribing will continue to gain traction in the Canadian context as funding initiatives and health care–community partnerships emerge from ongoing animated public discourse. As interest builds, it is important to critically examine this intervention strategy and caution against the too-rapid widespread adoption until evidence of effectiveness and best practices can be established. 

First, there is a need for further research on food prescribing in the Canadian context that incorporates large sample sizes, control groups and validated assessments of dietary intake, food security and health.^6^,^14^ Evaluations should also discern which program models (retail grocery vouchers vs. market vouchers vs. food boxes, delivery vs. local pick-up, and co-pay models vs. no-pay models) and which intervention components (subsidized food, nutrition education or dietetic counselling) have the greatest impact on patient outcomes.^22^ Researchers should also consider incorporating age and comorbidity subgroups. 

Meanwhile, process evaluations should identify how intervention models can be successfully integrated into existing primary care practice across multiple settings (including community health centres, family health teams, hospital and long-term care settings, and student health clinics). Given the administrative burden of food prescribing, it is unclear if this intervention model is more cost-efficient than others in addressing nutrition-related health outcomes. Health economics research is therefore needed to determine the relative efficiencies of various food security and public health nutrition program models, including food prescribing, to reduce health care utilization and spending. 

Access to health services, household food insecurity and nutrition-related chronic disease(s) are usually inclusion criteria for enrolment in food prescribing programs.^6^,^14^ It is unsurprising that, when provided with free or subsidized produce, individuals experiencing food insecurity at baseline consume more fruits and vegetables and experience improved food security over the course of a food prescription program.^14^ Yet, there is no evidence to suggest that benefits extend beyond the intervention period, as food prescriptions do little to address the underlying causes of food insecurity and dietary adequacy. As stated by Tarasuk and McIntyre, “Unlike policy interventions that reduce … food insecurity in the population by improving vulnerable households’ abilities to afford food … food prescription programs circumvent households’ financial constraints with respect to the purchase of food.”^22^^,p.2315^

Further, such programs may exclude individuals who do not access primary health care, those who are food insecure but not (yet) managing a chronic health condition, and those who are managing diseases that may be associated with food insecurity but not enforced as an inclusion criterion (e.g. mental health conditions). Food prescriptions that provide raw produce may also alienate potential participants with limited access to food preparation space and equipment. Such limitations threaten to exacerbate health inequities faced by populations experiencing food insecurity. Food prescribing therefore does not serve as a population-level solution to food insecurity and should not replace advocacy and policy initiatives that aim to address poverty and other structural determinants of food insecurity. 

There is also the question of paternalism. Food prescriptions usually provide food boxes or vouchers that can be spent on eligible healthy foods, serving as a conditional cash transfer but without the freedom of choice to spend funds on whatever is deemed necessary by the recipient (such as housing and other non-food basic necessities). If the health care system can provide food, would it be more dignified to prescribe cash? The “cash versus food” debate has played out in other disciplines,^23^ but with the advent of social prescribing, we must assess whether health care interactions are opportunities to provide targeted income support to address the social determinants of health. Preliminary evidence from FFRx suggests that most food prescription recipients preferred food prescriptions to cash, largely due to the quality of the food and the delivery service, which saved participants time and transportation costs. There may be additional benefits of food prescribing compared to income-based supports, including improved food literacy and interhousehold food sharing; however, the cash-versus-food question should be investigated further.

A pressing question that has seen little attention is how food prescribing fits into the various income and social support mechanisms that make up current social welfare systems. The failure of income support programs to eliminate poverty in Canada has resulted in a patchwork of programs designed to alleviate the constellation of challenges—from housing vulnerability to food insecurity—faced by low-income households.^24^ Food banks, which are independent, not-for-profit organizations relying on private donations and grants, are the most common model of food assistance in Canada.^25^ Food prescribing may address some of the limitations of food banks by leveraging health care interactions to provide targeted food assistance. Preliminary findings from our FFRx study suggest that users preferred food prescribing over food banks due to the convenience, overall food quality and experience of food prescribing as a less stigmatizing form of support.^26^

Moreover, FFRx users accessing income assistance (e.g. Ontario Works or Ontario Disability Support Program) reported that FFRx allowed them to spend cash previously allocated to food on other necessities.^26^ However, it remains to be seen if food prescribing is more effective than food banks at addressing longer-term food insecurity and nutrition among recipients and whether food prescribing is preferred to food banks by users more broadly (i.e. beyond our FFRx model that included the convenience of free delivery and online ordering). Further, without a source of sustainable (government) funding, food prescribing risks becoming yet another grant-funded model that is implemented by small networks of providers in an ad hoc and time-limited manner.^24^ In line with asset-based approaches to integrated care, there is a need to identify leverage points to build more robust practitioner–community partnerships and promote sustainable resource allocation to ensure the success of food prescribing programs. 

An additional finding to emerge from our research is the crucial importance of food prescription program design, including aspects of referral management, accessibility and de-stigmatization. Team-based care approaches and charting for food insecurity in electronic medical records can simplify the process of identifying and referring patients for food prescription programs. Once a food prescription has been provided, the way people use it can vary dramatically depending on the program model. We reported very high voucher redemption rates in the most recent phase of FFRx (participants redeemed over 88% of vouchers, in comparison to 34.5%–59% in most other studies), likely due to the program’s convenience (it included online ordering and free delivery); regular contact with Guelph CHC staff and researchers; and routine efforts to contact disengaged participants and address accessibility barriers.^6^,^27^ Such findings suggest that food prescribing requires concerted efforts to improve accessibility and provide robust support mechanisms and regular engagement between staff, health care providers and participants to maximize program utilization. These efforts clearly necessitate a high administrative burden and underscore the need to ensure primary care providers and community partners are equipped and adequately resourced before implementing large-scale, sustainable and effective food prescribing interventions in Canada. 

## Conclusion

Food prescribing is an innovative intervention model that shows promise for empowering health care providers to simultaneously address food insecurity, dietary adequacy and chronic disease management. As food prescribing gains momentum in Canada, it will be crucial to continue to gather and synthesize emerging evidence. While such programs may have a place in the Canadian health care and social welfare landscape, there is a need to critically evaluate their effectiveness and cost-efficiency relative to other health care, public health and social welfare programs. 

## Acknowledgements

Funding for the FFRx research project was received from the Sprott Foundation, the McConnell Foundation, Kindred Credit Union, The City of Guelph Community Fund, MAZON Canada, a Mitacs Accelerate Grant (no. IT26188) and a Canadian Institutes of Health Research Planning and Dissemination Grant (no. 478709). Matthew Little receives funding from a Michael Smith Health Research BC Scholar Award.

## Conflicts of interest

The authors declare no conflicts of interest.

## Authors’ contributions and statement

ML—conceptualization, funding acquisition, writing—original draft; writing—review and editing.

WD—conceptualization, writing—review and editing.

LJB—conceptualization, writing—review and editing.

AR—conceptualization, project administration, writing—review and editing. 

The content and views expressed in this article are those of the authors and do not necessarily reflect those of the Government of Canada.
